# Treatment of Third Branch Trigeminal Neuralgia With a Balloon Inflated in the Foramen Ovale

**DOI:** 10.3389/fneur.2022.826653

**Published:** 2022-02-23

**Authors:** Zhangfan Lu, Jiadong Wang, Yang Cao, Chenglong Sun, Quan Du, Yongfeng Shen, Wenhua Yu, Yuanfeng Du

**Affiliations:** ^1^The Fourth School of Clinical Medicine, Zhejiang Chinese Medical University, Hangzhou, China; ^2^Department of Neurosurgery, Affiliated Hangzhou First People's Hospital, Zhejiang University School of Medicine, Hangzhou, China

**Keywords:** trigeminal neuralgia, V3, modified, percutaneous balloon compression, functional neurosurgery

## Abstract

**Objective:**

To investigate the efficacy of modified percutaneous balloon compression for simple third branch pain and its postoperative complications.

**Methods:**

Clinical data and surgical records of 132 patients with third branch pain treated with percutaneous balloon compression from March 2015 to May 2019 were retrospectively analyzed, of which 81 cases were in the modified group and 51 cases were in the classic group. The modified technique was to compress again at the foramen ovale to enhance the compression in V3 after compression of the Gasserian ganglion.

**Results:**

In the modified group, the overall therapeutic efficiency was 96.3%, with 77 patients (95.1%) having immediate postoperative pain relief and one patient (1.2%) having occasional pain without the need for medication. In the classic group, immediate postoperative pain relief was seen in 43 cases (84.3%), and two patients (3.9%) had occasional pain with no need for medication. The rate of complete pain relief was significantly higher in the modified group than in the classic group (*P* < 0.05). Postoperative follow-up ranged from 14 to 48 months. The pain-free rates were 77.8 and 54.9% in the modified and classic groups, respectively. The incidence of facial numbness in the region of the first branch was significantly lower than in the classic group (*P* < 0.001).

**Conclusion:**

The modified procedure has significant advantages over the classic procedure in improving surgical efficacy, reducing postoperative recurrence rate, and decreasing postoperative numbness in the region, and can be used to treat simple trigeminal neuralgia in the third branch.

## Plain Language Summary

To date, few studies on percutaneous balloon compression (PBC) for third branch (V3) trigeminal neuralgia have been reported. This study aimed to investigate the efficiency, postoperative complications of the modified PBC for V3 pain. Our paper is the first to describe adequate compression of the V3 in the inner, outer foramina, and the channel of the foramen ovale. Our data show that the modified procedure has significant advantages over the classic procedure in improving surgical efficacy.

## Introduction

Percutaneous balloon compression (PBC) was first proposed by Mullan and Lichtor (1) in 1983. Due to its simplicity, short procedure, safety, and low pain occurrence when performed under general anesthesia, PBC is particularly suitable for patients of advanced age or with other underlying diseases who cannot tolerate major procedures. However, we found that the efficacy of PBC for third branch (V3) trigeminal neuralgia was relatively unsatisfactory, with a relatively increased area of postoperative numbness and a relatively high incidence of facial sensory abnormalities. This has also been reported by Frank and Fabrizi (2) and Teo and Suttner (3). Frank and Fabrizi believed that the unsatisfactory results were related to the high balloon position and insufficient compression of the third branch of the trigeminal nerve (2); thus, treatment using radiofrequency thermocoagulation is suggested for patients with third branch pain. To date, few studies on PBC for V3 have been reported in the literature. Therefore, this study aimed to investigate the efficiency, postoperative complications, and recurrence rate of the modified PBC for the treatment of third branch trigeminal neuralgia.

## Patients and Methods

### General Information

In this study, all patients had been approved by the appropriate ethics [Approval number: 2020(204)-01]. All procedures followed the Helsinki Declaration. Due to the retrospective nature of the study based on routine clinical procedures, informed consent was not required. All patients gave informed consent for procedures included in the study. Inclusion criteria: (1) A diagnosis of primary TN according to the International Headache Society (IHS) diagnostic criteria for TN, and Barrow Neurological Institute (BNI) pain class ≥ IV; (2) No remarkable positive neurological sign; (3) Trigeminal nerve-associated pain; (4) Fear of craniotomy or difficulty tolerating prolonged general anesthesia; (5) Difficulty tolerating medication (drug allergy or severe complications after medication). Exclusion criteria: (1) Secondary TN; (2) Medication efficient in the control of TN or BNI pain class < IV; (3) secondary surgery; (4) presence of progressive stroke and hydrocephalus; (5) surgical contraindications, such as severe seizure.

A total of 132 consecutive patients with trigeminal neuralgia on V3 branch, respecting the inclusion and exclusion criteria, were treated at our Institution with PBC (one patient died during follow-up, five patients were lost to follow-up, and these six patients were excluded from the statistical analysis) from March 2015 to May 2019. Of these, 51 patients, treated from March 2015 to November 2016, were submitted to a standardized PBC and were then considered as the classic group; on the contrary, 81 patients treated from December 2016 to May 2019 were submitted to a modified PBC (see below), and were then considered as the modified group. All patients did not respond to drug treatment, were allergic, or had severe complications. None had undergone other invasive surgical treatment before the surgery. Modified group: 35 males and 46 females; age 35–82 years, mean age 59.3 ± 5.4 years; 36 left-sided and 45 right-sided. Classic group: 23 males and 28 females; age 32–86 years, mean age 60.5 ± 3.9 years; 27 left-sided and 24 right-sided. Preoperative cranial computed tomography (CT) or magnetic resonance imaging was performed to exclude secondary trigeminal neuralgia, and 3D reconstruction of conventional skull base CT was performed to understand the anatomical structure of the foramen ovale.

### Surgical Method

#### Classic Group

**Figure 1:**
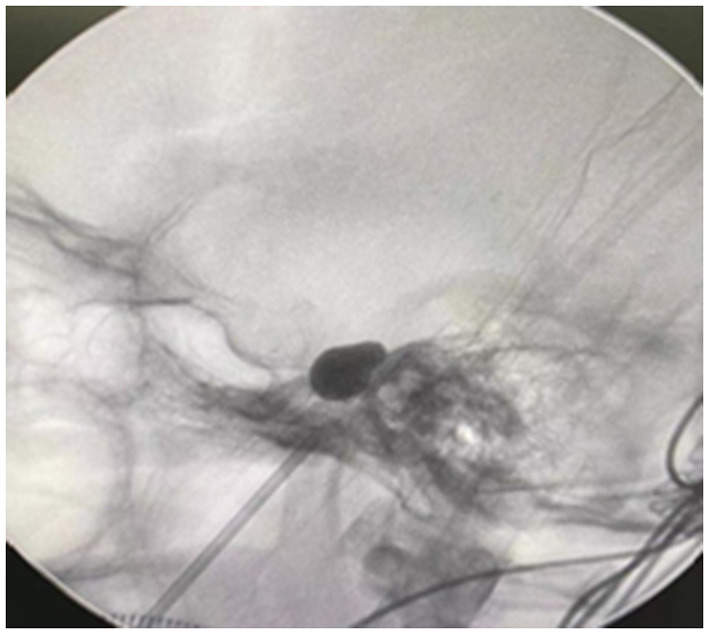
The inflated balloon with the characteristic pear shape of the balloon indicating the position of the balloon within the porus trigeminus.

The patients were placed supine after intubation under general anesthesia with slight extension and neck rotation of ~30° away from the affected side. The Hartel anterior trigeminal nerve Gasserian ganglion puncture technique consistent with conventional PBC was used, with the entry point selected ~2–3 cm outside the affected corner of the mouth (point A), about 3 cm in front of the external auditory canal at the level of the zygomatic arch (point B), and around 1 cm below the ipsilateral pupil (point C). The puncture was extended from A in the direction of AB and punctured inward and upward toward point C. A 14-gauge blunt-tipped core puncture needle was used to place the puncture needle in the foramen ovale with reference to the temporal petrous bone, sella turcica, and clivus, and other bony landmarks next to the foramen ovale under the surveillance of the X-ray C-arm machine. A No. 4 Forgarty balloon was placed in the Meckel cavity, and ~0.3–0.6 ml of contrast agent Omnipaque was slowly injected into the balloon while the position and shape of the balloon were checked. Intraoperatively, if the balloon shape was not satisfactory, the balloon was immediately emptied and repositioned until the desired standard pear shape appeared (Figure 1). After compressing the Gasserian ganglion for ~3–4 min, the balloon was immediately emptied, the catheter was removed, and the puncture site was compressed for 5–10 min.

#### Modified Group

**Figure 2:**
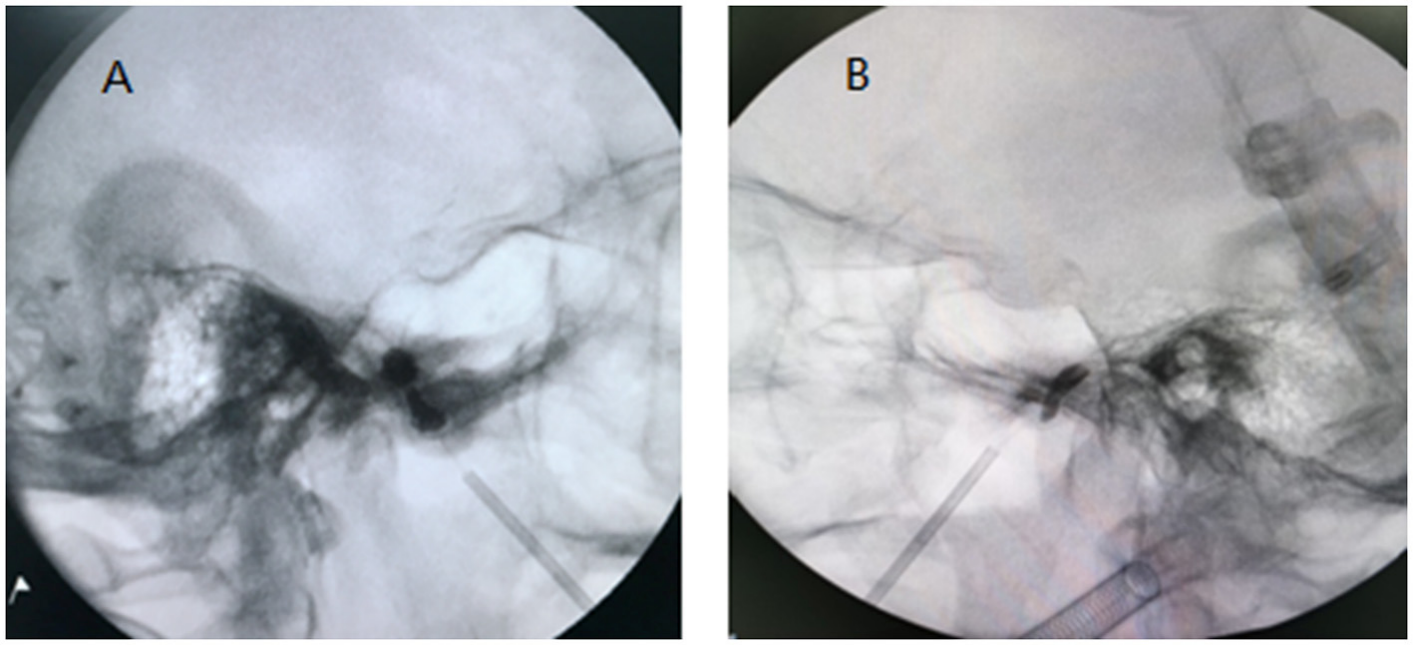
The Fogarty balloon catheter located at the entrance of the foramen ovale. Inflating the balloon with dumbbell shape **(A)** or sausage shape **(B)**. Now the balloon could increase pressure on V3.

The procedure for compressing the Gasserian ganglion was the same as in the classic group. After 1–2 min of compression, the balloon was emptied, the balloon catheter was removed and placed at the entrance of the foramen ovale, and ~0.1–0.2 ml contrast agent Omnipaque was slowly injected to compress the balloon, which is preferably dumbbell-shaped or sausage-shaped (Figure 2). Compression was performed for 1–2 min. If the balloon bursts, the contrast should be promptly removed and the balloon replaced. Postoperative facial compression should be done as previously described.

### Postoperative Outcome Evaluation and Follow-Up

All patients were followed up postoperatively by the neurosurgeon in charge to observe the postoperative outcomes. The postoperative outcome was assessed using the Barrow Neurological Institute (BNI) prognostic grading (Table 1). Grade I pain was considered as cured, and grade I + II was considered as an effective treatment.

**Table 1:** Barrow Neurological Institute (BNI) pain intensity scale score.

**Score**	**Definition**
I	No pain, no medication
II	Occasional pain, not requiring medication
III	Some pain, adequately controlled with medication
IV	Some pain, not adequately controlled with medication
V	Severe pain/no pain relief

### Statistical Analysis

The IBM SPSS 21.0 software package was used for statistical processing. The cure rate, incidence of postoperative complications, and recurrence rate were analyzed using the chi-square test, and postoperative pain-free survival was analyzed using the Kaplan-Meier method.

## Results

### Postoperative Efficacy

**Figure 3:**
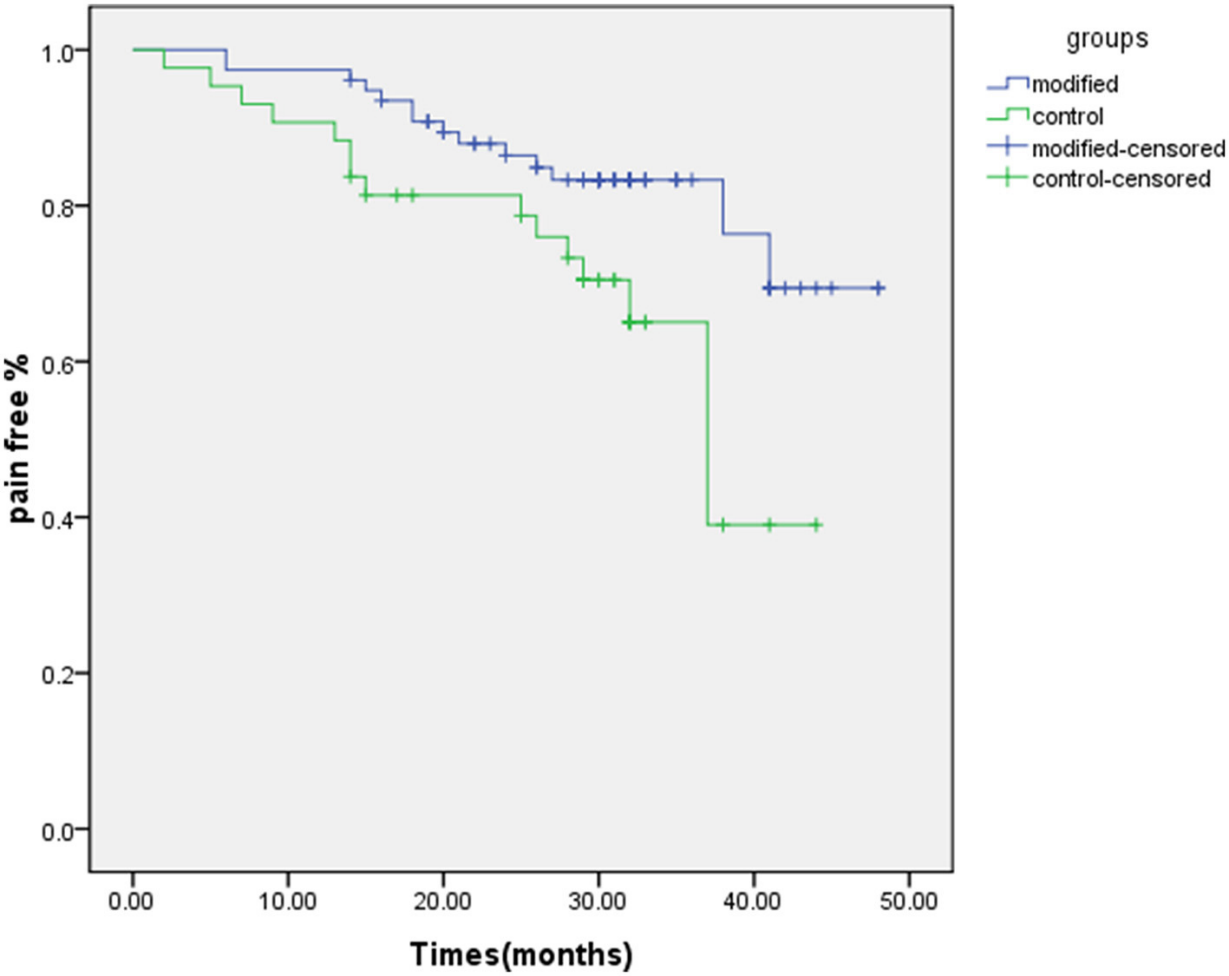
Kaplan-Meier survival curve showed that the postoperative pain-free maintenance period was significantly higher in the modified group than in the classic group (*P* = 0.025). The mean follow-up was 30.2 months.

Immediate postoperative pain relief (BNI class I) was observed in 77 (95.1%) of the 81 patients in the modified group, and one (1.2%) patient had occasional pain without medication needed (BNI class II), with an overall treatment efficiency of 96.3%. In the classic group, immediate postoperative pain relief (BNI class I) was seen in 43 (84.3%) of 51 patients, and two (3.9%) patients had occasional pain without medication needed (BNI class II), with an overall treatment efficiency of 88.2%. The rates of complete pain relief and treatment efficiency in the modified group were significantly higher than in the classic group (*P* < 0.05). Long-term postoperative follow-ups were performed for the patients, with a follow-up period of 14–48 months and a mean of 30.2 months. At the time of final follow-up before additional surgical procedures, the pain-free rates were 77.8 and 54.9% in the modified and classic groups, respectively. Kaplan-Meier survival analysis (Figure 3) showed that the postoperative pain-free maintenance period was significantly longer in the modified group than in the classic group (*P* = 0.025). Intraoperative balloon rupture occurred in eight cases (9.9%) in the modified group and two cases (3.9%) in the classic group. Moreover, eight patients with recurrence in the modified group and ten patients in the classic group were re-treated with the modified PBC at our center and no pain recurrence at the subsequent follow-up. The follow-up time of the recurrent patients was calculated from the time after PBC to the recurrence of pain, and for the others, it was calculated from the PBC to the last follow-up.

### Postoperative Complications

Table 2 shows postoperative complications in both groups. Facial numbness in the region of the ipsilateral V3 was the most common complication and was mostly tolerated. The incidence of postoperative facial numbness in the region of V3 was significantly higher in the modified group than in the classic group (*P* = 0.013), 98.8 and 88.2%, respectively. The incidence of postoperative facial numbness in the region of the first branch of the trigeminal nerve (V1) was significantly lower in the modified group than in the classic group (*P* < 0.001), 14.8 and 70.6%, respectively. There was no significant difference between the two groups in the incidence of postoperative complications, such as abnormal sensation, masseter muscle weakness, herpes simplex, and diplopia. No serious complications occurred, such as keratitis complications, internal carotid artery cavernous sinus fistula, and intracranial subarachnoid hemorrhage. Patients with paresthesia presented with tingling sensations, of which three recovered within 8–15 months. Masseter muscle weakness mostly improved or returned to normal in 3–6 months, and most of the herpes of the mouth and lips recovered within 2 weeks.

**Table 2:** The complications of the two groups.

**Complications**	**Modified group (%)**	**Classic group (%)**	***P***
Facial numbness (V3)	80 (98.8)	45 (88.2)	0.013
Facial numbness (V1)	12 (14.8)	36 (70.6)	<0.001
Masseter muscle weakness	50 (61.7)	29 (56.9)	0.354
Herpes simplex	37 (45.7)	22 (43.1)	0.458
Paresthesia	4 (4.9)	2 (3.9)	0.573
Diplopia	0 (0.0)	1 (2.0)	0.386

The average length of hospitalization was 2–3 days in both groups. The average operative time in the classic group was 14–19 min. The operative time in the modified PBC group was 18–26 min.

## Discussions

In PBC treatment, the shape of the balloon in a typical pear shape intraoperatively is now widely recognized as the key to successful PBC (4–8). In this study, in patients with pain in the third branch, after 1–2 min of compression of the Gasserian ganglion, the balloon was compressed again for 1–2 min with a 0.1–0.2 ml-sized balloon at the foramen ovale to enhance the compression in V3. Longer compression times were used only if the patient had multiple invasive surgical treatments, recurrent pain, or severe long-term pain. Patients with a history of previous surgery were excluded from this study.

According to several studies (9, 10), the most common site of trigeminal neuralgia is the V3 or second branch (V2) + V3. Due to the marginal anatomical location of the V3, if the intraoperative balloon volume of PBC is too small, the V3 is compressed insufficiently, causing relatively unsatisfactory postoperative outcomes and a high postoperative recurrence rate. If the intraoperative balloon volume is >0.6 ml, V1 and V2 are susceptible to overcompression by the oversized balloon, resulting in postoperative numbness in their innervated areas and an increased incidence of facial sensory abnormalities. Postoperative facial numbness is a positive indicator, suggesting that the balloon provides effective compression and, thus, a longer period of pain-free survival (2). In this study, the incidence of postoperative facial numbness in the V3 region was significantly higher in the modified group than in the classic group (*P* = 0.013), suggesting a more effective compression effect on V3.

Cheng et al. (11) suggested that the efficacy of the balloon was more pronounced for V3 when it was located lateral to the Meckel's cavity. In our study, when the balloon catheter was slightly backed up to the entrance of the foramen ovale after compressing the Gasserian ganglion, the balloon was filled again to a dumbbell or sausage shape at this time. Adequate compression of the V3 was performed in the inner and outer foramina of the foramen ovale and the channel of the foramen ovale. There was no obvious effect on V1 and V2. However, we found that the balloon was prone to rupture at this time, and more practice is required to optimize this operation, with a filling of 0.1–0.2 ml being preferred (determined by the size of the individual foramen ovale) and a compression time of 1–2 min.

In this study, the modified procedure targeted the anatomical structure of the V3 and provided effective compression of both the internal and external foramina of the foramen ovale and V3 within the foramen ovale channel, controlling the balloon size between 0.1 and 0.2 ml without significant compression in V1. The incidence of postoperative numbness in the region of V1 was 14.8% in the modified group, which was significantly lower than in the classic group (70.6%). The incidence of postoperative facial numbness in the region of V3, the rate of complete pain relief, and treatment efficiency were significantly higher in the modified group than in the classic group. The balloon was prone to rupture during this secondary compression operation, and no significant adverse effects were found in the postoperative follow-up of patients with balloon rupture due to the use of nonionic contrast. Due to effective intraoperative control of balloon size, there were no significant differences in the incidence of postoperative facial sensory abnormalities between the two groups. In the postoperative follow-up observation, the postoperative pain-free maintenance period was significantly longer in the modified group than in the classic group, indicating that the modified procedure can safely provide effective compression of V3, which is significant for improving efficacy, reducing the area of postoperative facial numbness, and decreasing the postoperative discomfort of patients. Compared with the preoperative nerve pain, severe postoperative facial numbness of the patients, masseter muscle weakness, and paresthesia also seriously impacted the quality of life of the patients.

Teo and Suttner (3) reported the use of a handheld traction device for the treatment of pain in the third branch. Intraoperatively, when the balloon was in a typical pear shape, the use of the handheld traction device caused a smaller displacement of the filled balloon toward the foramen ovale, providing an enhanced compression of V3 in the internal foramen portion of the foramen ovale. This was more effective in compressing V3 compared to the no-traction group, and its therapeutic efficiency was better than that of the no-traction group. In the modified procedure of this study, the balloon provided effective compression of V3 in both the internal and external foramina of the foramen ovale and the foramen ovale channel. The overall treatment efficiency was 96.3% in the modified group and 88.2% in the classic group. The postoperative pain relief rate was significantly higher in the modified group than in the classic group (*P* < 0.05). After a mean follow-up of 30.2 months, the pain-free maintenance period was significantly longer in the modified group than in the classic group, at 77.8 and 54.9%, respectively. The V3 trigeminal neuralgia also currently has the option of radiofrequency thermocoagulation (RF) for treatment, and this study provides a novel attempt. Sterman-Neto et al. (12) showed similar overall efficacy of PBC compared to RF in terms of analgesia, but dysesthesia due to PBC was less common. PBC has a shorter learning curve and lower cost, so it is preferentially recommended to use PBC for trigeminal neuralgia. The modified technique proposed in this paper can be further compared with RF treatment of the V3 trigeminal neuralgia in the future to investigate the efficacy between the two percutaneous procedures.

## Conclusion

For patients with third branch trigeminal neuralgia, the modified PBC technique has significant advantages over the conventional PBC technique in terms of improved surgical efficacy, reduced postoperative recurrence rate, and reduced postoperative numbness in the area. This modified PBC procedure could be a safe and effective method of treating simple third branch trigeminal neuralgia.

## Limitation

The current study has several limitations. First, this is a single-center retrospective study, and multi-center randomized controlled trials with a larger sample size are required to prove the current study's finding further. Second, the follow-up period of the two groups was short, and the long-term complications and recurrence might not be sufficiently assessed. The bias caused by the difference in follow-up period between the two groups cannot be excluded.

## Data Availability Statement

The raw data supporting the conclusions of this article will be made available by the authors, without undue reservation.

## Ethics Statement

The studies involving human participants were reviewed and approved by Affiliated Hangzhou First People's Hospital, Zhejiang University School of Medicine, Hangzhou, China. Written informed consent for participation was not required for this study in accordance with the national legislation and the institutional requirements.

## Author Contributions

ZL and JW drafted the manuscript. WY and CS participated in surgical treatment. QD participated in the design of the study and performed the follow-up. YC and YS helped to sort data. WY and YD conceived of this study, participated in its design and coordination, and helped to draft the manuscript. All authors read and approved the final manuscript.

## Funding

This work was supported by the Zhejiang Medicine and Health Technology Project (Grant Number 2021KY229).

## Conflict of Interest

The authors declare that the research was conducted in the absence of any commercial or financial relationships that could be construed as a potential conflict of interest.

## Publisher's Note

All claims expressed in this article are solely those of the authors and do not necessarily represent those of their affiliated organizations, or those of the publisher, the editors and the reviewers. Any product that may be evaluated in this article, or claim that may be made by its manufacturer, is not guaranteed or endorsed by the publisher.
